# Undernutrition and Mortality among Adult Tuberculosis Patients in Addis Ababa, Ethiopia

**DOI:** 10.1155/2020/5238010

**Published:** 2020-07-27

**Authors:** Getachew Seid, Marta Ayele

**Affiliations:** Ethiopian Public Health Institute, Addis Ababa, Ethiopia

## Abstract

**Background:**

In developing countries, there are several adult tuberculosis (TB) patients suffering from profound undernutrition. Undernutrition is a significant risk factor for developing tuberculosis. In the world, TB is one of the top ten and leading causes of death. To appropriately intervene death of adult TB patients, it is crucial to understand the magnitude of undernutrition and its associated factors among them. The study assessed undernutrition and mortality among adult tuberculosis patients in Addis Ababa, Ethiopia.

**Methods:**

Institutional-based retrospective study was conducted in Addis Ababa, Ethiopia, from January 2019 to August 2019. The total sample size of the study was 284. The source populations were TB patients who have followed up for TB treatment at public health facilities of Addis Ababa. The sample size was allocated to the selected health facilities proportional to their size, and study subjects were enrolled to the study during the study period. Data were collected by a structured data sheet from the selected health center registration book. Data were entered into Epi Data software and analyzed by using SPSS version 20. Descriptive statistical methods were used to summarize the sociodemographic characteristics of the study participants. Survival curves were generated using the Kaplan–Meier method for all TB patients.

**Result:**

A total of 284 study participants were included in the study. It was found that 46.8% of the study population have undernutrition (BMI <18.5 kg/m^2^) at the time of registration for treatment. Out of undernourished patients, 54 (19.0%) had severe malnutrition and 78 (27.5%) had moderate undernutrition. At the end of the two-month intensive treatment period, the under nutrition prevalence declined to 38.7%. Of the 284 patients, 17 (6.0%) died before completing anti-TB treatment. Three quarters of all forms of TB deaths occurred within 57 days after the start of anti-TB treatment. The proportion of deaths by nutritional status at treatment initiation among normal, moderate acute malnutrition, and severe acute malnutrition TB patients was 3.1%, 8.9%, and 16.3%, respectively.

**Conclusion:**

Almost half of the TB patients were undernourished at the start of anti-TB treatment based on BMI. From the malnourished, less than 20% of the participants gained weight and moved to normal weight at the end of the two-month intensive treatment period. The high death rate was reported among severely malnourished tuberculosis patients, but it needs a larger study to further understand predictors. To enhance the increment of nutritional status during treatment, the government should give attention to support nutritional supplements for TB patients.

## 1. Background

In the world, from a single infectious agent, above HIV/AIDS, tuberculosis (TB) is one of the top ten and leading causes of death. There were an estimated 1.2 million (range, 1.1–1.3 million) TB deaths among HIV-negative people in 2018, and an additional 251,000 deaths (range, 223,000–281,000) among HIV-positive people [1]. There were TB reports from all countries and age groups; from this, 90% were adults (aged ≥ 15 years) and 9% were people living with HIV (72% in Africa). Globally by 2030, the ambition is to reduce TB mortality by 90% compared with 2015 [2]. Among the 22 highest tuberculosis- (TB-) burdened countries, Ethiopia ranks third in Africa and eighth in the world. The incidence rate of the total TB rate is 151 (107–204) per 100,000 population. The TB case detection rate, the treatment success rate, and the TB cure rate were 74%, 82.5%, and 67%, respectively [1]. In Ethiopia, the estimated TB mortality in 2019 stood at 24 (15–36) cases per 100,000 population [1].

Although undernutrition, HIV infection, diabetes, and cancer were the known risk factors for active tuberculosis, undernutrition has the highest population attributable fraction of 27% [3]. The risk of death can be possibly reduced through the understanding of mortality predictors and by improving patient care [4, 5].

There were globally 2.3 million new TB cases in 2018 that were attributable to undernutrition [1]. In Ethiopia, HIV and TB infections are important contributing factors to malnutrition [6]. In developing countries, there are several adult tuberculosis (TB) patients suffering from profound undernutrition [7, 8]. It is estimated that undernutrition causes about one-quarter of all new TB cases globally [9]. This can have serious public health impacts if those with undernourished adult TB are not identified early [10]. The relationship between TB and undernutrition is bidirectional because TB disease leads to secondary undernutrition and vice versa [11].

Persons at high risk for developing TB disease fall into two categories: persons who have been recently infected with TB bacteria and persons who were immunocompromised, such as lower body weight [12]. In the clinical course of the disease, most individuals with active TB experience loss of appetite, nausea, and abdominal pain, which reduce food intake and lastly cause weight loss. Equally, undernutrition weakens the body's ability to fight disease. So undernutrition increases the likelihood that latent TB will develop into active TB disease [13].

A less than ideal weight gain during TB treatment also increases the risk of long-term relapse even after the initial cure. Undernutrition has also been associated with malabsorption of key anti-TB drugs [13]. To appropriately intervene in the nutritional problems of adult TB patients, it is crucial to understand the magnitude of undernutrition and its associated factors among them [14, 15]. A study conducted in Addis Ababa [16] found that the prevalence of undernutrition was 39.7%.

To improve treatment outcomes of TB patients, understanding the risk factors for TB mortality and undernutrition plays a crucial role in finding out strategies and interventions to solve the problem. Even though in Ethiopia there was a high burden of TB and undernutrition, only a few studies have been conducted regarding the mortality and associated factors of undernutrition among TB patients particularly in urban settings. Therefore, the main objective of this study was to assess undernutrition and mortality among tuberculosis patients in Addis Ababa, Ethiopia.

## 2. Methods

### 2.1. Study Settings

This study was conducted in Addis Ababa, the capital city of Ethiopia. According to the 2007 Ethiopian central statistics census, Addis Ababa is the biggest city in the country by population and area, with a total population of 3,384,569. This capital city occupies 527 square kilometers of a region in Ethiopia. The population density is estimated to be approximately 5165 individuals per square kilometer available [17].

### 2.2. Study Design and Participants

An institutional-based retrospective study was carried out in Addis Ababa, Ethiopia, from January to August 2019 to evaluate undernutrition and any form of mortality among tuberculosis patients. The target populations were TB patients who were attended for TB treatment at public health facilities of Addis Ababa. However, the study populations were TB patients aged 18 years and above who have complete data on the registration books of health facilities. The patients treated with ethambutol (E), rifampicin (R), isoniazid (H), and pyrazinamide (Z) during the intensive phase of two months, followed by four “months” (1 “month” = 4 weeks) with ethambutol and isoniazid: 2ERHZ/4EH; the daily dosage of the drugs combination depends on the weight of the patient.

### 2.3. Study Variables

Sociodemographic and other variables such as weight at treatment initiation, weight at the end of second-month treatment, HIV status, TB type, treatment outcome, and others were extracted from the documents of all TB cases in three randomly selected health centers.

### 2.4. Definition of Variables

Pulmonary TB (PPOS) included a patient with bacteriological (by microscopy or GeneXpert) confirmed TB. Pulmonary TB, smear-negative (PTB-) included a patient with symptoms suggestive of TB, which were negative by microscopy or GeneXpert and with chest radiograph abnormalities consistent with active pulmonary TB. Extrapulmonary TB (EPTB) included tuberculosis of organs other than the lungs [18].

Body mass index is the person's weight in kilogram (kg) divided by his or her height in metres squared and used to determine the nutritional status of TB patients and classified as follows: severe undernutrition (BMI < 16.0 kg/m^2^), moderate undernutrition (BMI = 16.0–18.49 kg/m^2^), normal weight (BMI = 18.5–24.99 kg/m^2^), and overweight (BMI  greater than  25.0 kg/m^2^). In order to measure the weight, the patients were asked to stand with bare foot on the center of balance and the weight was recorded to the nearest 0.1 kilogram. By the same procedure, the height was measured by asking the patient to be barefoot, wearing no headgear, knees fully straight, and both hands held down to the side, and the height was recorded to the nearest 0.5 centimeters.

The treatment outcome variable was recorded as (a) cured (finished treatment with negative bacteriology result at the end of treatment), (b) treatment completed (patient finished treatment but no bacteriology result at the end of the treatment), (c) treatment failure (smear-positive at five months despite correct intake of medication), defaulter (patients who interrupted their treatment for two consecutive months or more after registration), and died (patients who died from any cause during treatment), (d) transferred out (patients whose treatment results are unknown due to transfer to another health facility), and (e) successfully treated (the sum of patients who are declared “cured” and those who have “completed” treatment). Survival time was defined as the time in days from the beginning of treatment to death from tuberculosis as the main or associated cause. Censoring occurred either at the end of the study or due to death from other causes and include transfer outs [18].

### 2.5. Sampling Procedure

The African center, Addis Ababa city administration, divides into ten subcities. Based on their health service coverage, we divided the subcities into low, medium, and high; from each group, one health center, which provides anti-TB service (DOTS), was randomly selected by listing all available health centers in each group.

### 2.6. Data Collection and Quality Assurance

The registration documents of each DOT clinic contain the basic information of patients such as demographic, treatment history, diagnosis, anthropometric data, and treatment started dating, follow updates, coinfection, household contact number, treatment outcomes, and other data. Data were collected by a structured data sheet from the selected health center registration book. Data were extracted from the registration book by well-trained clinical nurses working in other unselected health centers TB clinics.

To ensure the quality of the collected data, one-day training was given to data collectors on the objective, methods, tool, and ethics of the study. The overall activity was monitored by the principal investigator and coinvestigators daily.

### 2.7. Data Management and Analysis

Data were entered into Epi Data software and analyzed by using SPSS version 20. The level of significance was set at 0.05. Descriptive statistical methods were used to summarize the sociodemographic characteristics of the study participants. Survival curves were generated using the Kaplan–Meier method for all TB patients.

### 2.8. Ethical Approval

Before the study was conducted, ethical approval was obtained from Addis Ababa health bureau ethical review board. The protocol approved by Addis Ababa health bureau ethical review board noted that the data were collected as secondary data.

## 3. Results

Two hundred eighty-four (284) adult TB patients were enrolled during the study period. Of these, 284 participants, the majority (37.3%) of them, were between 25 and 34 years of age, with the mean age being 36.19 years (SD ± 15.99). More than half (56.4%) of them were males. Most (90.5%) were predominantly new tuberculosis patients. One hundred and forty (49.3%) were registered as bacteriologically confirmed pulmonary TB and 59 (20.8%) as smear-negative pulmonary TB patients (Table 1).

The study participants mean BMI at the initiation of treatment was 19.24 kg/m^2^; for males and females, it was 18.84 kg/m^2^ and 19.76 kg/m^2^, respectively. One hundred thirty-one patients (46.5%) were undernutrition at the time of registration; 54 (19.0%) having severe undernutrition and 78 (27.4%) moderate undernutrition. Among the total study participants, 51 (17.9%) had HIV coinfection (Table 2).

At the time of registration, undernutrition status was significantly associated with age group, sex, tuberculosis type, number of household members, weight group, and height group (Table 3).

Two months after the start of intensive phase treatment, there was a gradual improvement in nutritional status. The mean BMI for all patients increased to 20.0 kg/m^2^, a change of 4.4%. For males, the mean BMI rose to 19.84 kg/m^2^ and 20.2 kg/m^2^ for females, representing changes of 5.0% and 2.4%, respectively. One hundred and ten patients (38.7%) were malnourished; 40 (14.1%) had severe malnutrition, and 70 (24.60%) had moderate malnutrition.

By the end of the two months intensive phase of treatment, BMI increased in 183 (64.6%) patients, decreased in 65 (22.9%), and did not change in 35 (12.3%) of the patient. Change in BMI was significantly associated with the age group (Table 4).

### 3.1. Survival Analysis of TB Patients

**Figure 1:**
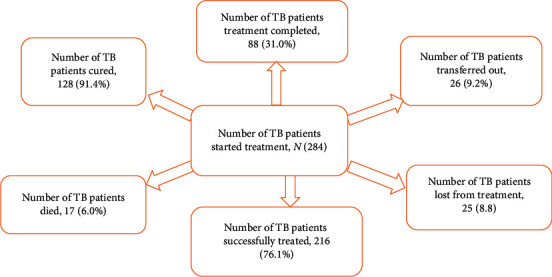
Treatment outcome of tuberculosis patients in Addis Ababa, 2019.

Of the 284 patients, 17 (6.0%) died before completing anti-TB treatment. Their mean age was 36 (range: 18–85) years. Three quarters of all forms of TB occurred within 57 days after the start of anti-TB treatment. A total of 284 participants was followed for a total of 48,901 person-days. Median survival for patients who died of TB was 44 days (range: 21–91) (*p* < 0.001 by the log-rank test). Only around three quarters (76.1%) of the patients were successfully treated (Figure 1).

**Figure 2:**
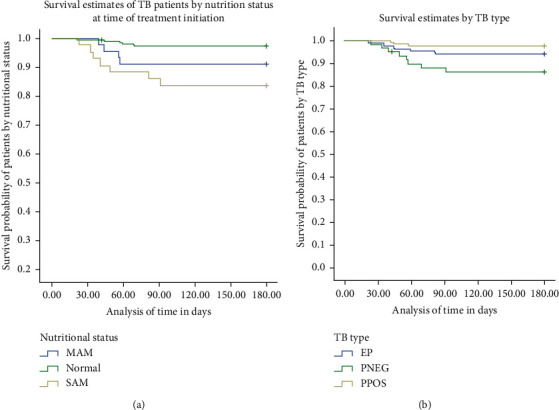
The survival curve of TB patients by (a) nutritional status at start of treatment and (b) type of tuberculosis in Addis Ababa, 2019.

In this study, the proportion of deaths by nutritional status at treatment initiation as normal, MAM, and SAM TB patients was 3.1%, 8.9%, and 16.3%, respectively. According to this study, second-month nutritional status, height, being HIV tested, sex, and type of TB were significantly associated with treatment outcome (*p* value <0.05). By the end of two-month anti-TB treatment, the survival probabilities unlikely of their TB type were 88%, 91% and 97% among SAM, MAM, and normal TB patients, respectively (Figure 2).

## 4. Discussion

This study revealed that around half of TB patients were undernutrition at the time of registration for anti-TB treatment. Moreover, second-month nutritional status, height, being HIV tested, sex, and type of TB were significantly associated with the treatment outcome of patients.

This finding of the result was similar to result reported from Ghana by Ea dodor in 2008 [19]. This might be because Ghana and Ethiopia were developing countries with proportional socioeconomic status. Before starting anti-TB medications, the patient has not recovered from their illness and was at the state of critical undernourishment, which could overestimate the expected figure of undernutrition. Moreover, most of the patients come to health centers after a long time of illness, which causes weight loss. But the result was high relative to previous studies conducted in Nepal with a prevalence of 36.1% [20] and lower than the results from studies conducted in India, Brazil, Malawi, and Tanzania with a prevalence of more than 85%, 70.6%, 57%, and more than 58%, respectively [21–24]. It might be due to the difference in sampling size and study setting. Additionally, socioeconomic and food intake habits of the study population were different.

Compared to this study, a study conducted in Gondar and Sidama reported a higher prevalence of undernutrition, 65.4% [25] and 77.9% [26], respectively. The study in Gondar had included the high number of TB/HIV coinfected patients who were more exposed to undernutrition because of a double burden. Additionally, it might be due to the difference in the study area (urban TB patients in our case), and in most of Ethiopian household, food intake patterns was at least three times per day in urban and two times per day in the rural population.

TB/HIV coinfection in this study (17.95%) was lower than previous reports from Adama (25.0%) [27] and Gondar University Hospital (52.1%) [28], respectively, but higher than studies such as Nekemte (11.5%) [29] and West Arsi (13.6%) [30]. According to the Ethiopian Public Health Institute 2018 report, Adama city was the hot spot area for HIV next to Gambella and Addis Ababa.

By the end of the two-month intensive phase of treatment, there was a gradual improvement in nutritional status (38.7% undernourished). Improvement in nutritional status with the commencement of treatment has been demonstrated in other studies [24, 31]. However, this improvement was only due to treatment, and besides anti-TB treatment, nutritional supplementation of TB patients will help to improve their status in a shorter time. Food supplementation of TB patients needs adequate political commitment, funding, and monitoring.

In the age group of 25–34, more than two-thirds (70.7%) of TB patients had weight gain at the end of two months of treatment. This may be due to the immune system of the young which is generally stronger, meaning the ability of the young to withstand and to recover from the stress of sickness is better than the aged.

This study has identified a low case mortality rate among a cohort of TB patients on treatment in Addis Ababa. The majority of deaths in this cohort occur two months after the initiation of anti-TB treatment. We have compared treatment outcomes across different risk factors. Factors significantly associated with mortality included a second-month nutritional status, height, sex, and type of TB.

Mortality among patients on TB treatment reported from Addis Ababa is lower (3.7%) [32] than figures found in this study (6.0%), but a similar result was discovered from south India [33]. The difference might be possibly due to the sample size difference.

From the total death, three-fourths occurred within the two-month intensive period treatment course, which was consistent with a study report from England [34] and Australia [35]. Early mortality after treatment onset reflects advanced disease and could be attributed to delayed treatment and late diagnosis [36, 37], disease progression of additional comorbid illnesses, drug toxicity, and poor adherence [38, 39]. Most of the patients came to health facilities after they tried to improve their health problems with cultural drugs either by lack of awareness about free TB diagnosis service or another factor.

The high death rate showed in this study among severely acute malnourished (SAM) TB patients might be because severely ill patients lose their weight, which implies that their immunity was subsidized. This causes difficulty for them to battle the side effects of the drug and the comorbidities. In addition to supply micronutrients to patients, early TB diagnosis and treatment may decrease the mortality rate.

## 5. Strengths and Limitations of the study

The study has pointed an important area of research interest which could be an input for the prevention of health problems related to undernutrition in adult TB patients, and the study has indicated the magnitude of undernutrition in the urban setting of the country. In the case of limitations, the study may not be generalized to other parts of the country because of the difference in sociodemographic and economic situations. The difficulty in ascertaining whether it was undernutrition that led to the development of TB or TB led to malnutrition was still in the study. Due to the shortage of vitamin D tests, we could not test the patients for vitamin D deficiency. The third was that we only use BMI for measurement of undernutrition. Lastly, the analyses included all deaths, irrespective of the cause of death.

## 6. Conclusion

Almost half of the TB patients were undernourished at the start of anti-TB treatment based on BMI. From the malnourished, less than 20% of the participants gained weight and moved to normal weight at the end of the two-month intensive treatment period. The high death rate was reported among severely malnourished tuberculosis patients, but it needs a larger study to further understand predictors. To enhance the increment of nutritional status during treatment, the government should give attention to support nutritional supplements for TB patients.

## Figures and Tables

**Table 1:** Sociodemographic characteristics of TB patients (*N* = 284).

Variable		*N* (%)
Age group	18–24	64 (22.5)
	25–34	106 (37.3)
	35–44	43 (15.1)
	45–60	40 (14.1)
	>60	31 (10.9)

Mean = 36.19, SD = 15.99, min = 18, and max = 88		

Sex	Male	160 (56.4)
	Female	124 (43.7)

TB type	PPOS	140 (49.3)
	PTB-	59 (20.8)
	EP	85 (29.9)

Treatment history	New	257 (90.5)
	Relapse	26 (9.5)

Linked by	PHF	232 (81.7)
	PPM	39 (13.7)
	HEW	13 (4.6)

GeneXpert tested	Yes	132 (46.5)
	No	152 (53.5)

PHF, linked directly to the public health facility by themselves; PPM, linked to a TB clinic through public private members (NGOS and private clinics); HEW, linked to a TB clinic through a health extension worker; PPOS, bacteriologically confirmed pulmonary tuberculosis patient; PTB−, pulmonary tuberculosis patient with only chest radiography finding; EP, extrapulmonary tuberculosis patients.

**Table 2:** Anthropometric and related information about tuberculosis patients at the start of treatment.

Variable		*N* (%)
Weight group in kg	<50	136 (47.9)
	50–60	90 (31.7)
	>60	58 (20.4)

Mean = 52.59, SD = 10.19, mini = 28, and max = 99		

BMI group in kg/m^2^	<16.0	54 (19.0)
	16.0–18.49	78 (27.4)
	18.5–24.9	127 (44.3)
	>24.9	25 (8.8)

Mean = 19.24 kg/m^2^, SD = 3.85, mini = 9.92 kg/m^2^, and max = 38.6 kg/m^2^		

Height group in metres	<1.60	102 (35.9)
	1.60–1.75	146 (51.4)
	>1.75	36 (12.6)

Mean = 1.64 metres, SD = 0.09, mini = 1.45 metres, and max = 1.96 metres		

HIV result	Positive	51 (17.9)
	Negative	233 (82.0)
Number of household members	0–3	200 (70.4)
	>3	84 (29.5)

**Table 3:** Factors associated with the nutritional status of the patient at the beginning of the treatment.

Variable		BMI <16.0, *N* (%)	BMI 16.0–18.49, *N* (%)	BMI 18.5–24.9, *N* (%)	BMI >24.9, *N* (%)	*p* value	AOR (95% CI)
Age group	18–24	11 (17.1)	25 (39.0)	28 (43.7)	0 (0)	0.001	0.65 (0.53–0.75)
	25–34	22 (20.7)	27 (25.4)	51 (48.1)	6 (5.6)		
	35–44	8 (18.6)	11 (25.5)	21 (48.8)	3 (6.9)		
	45–60	4 (10.0)	8 (20.0)	16 (40.0)	12 (30.0)		
	>60	9 (29.0)	7 (22.5)	11 (35.4)	4 (36.3)		

Sex	Male	33 (20.6)	46 (28.7)	74 (46.2)	7 (4.37)	0.028	0.31 (0.28–0.46)
	Female	21 (16.9)	32 (25.8)	53 (42.7)	18 (14.5)		

Treatment history	New	45 (17.5)	72 (28.0)	117 (45.5)	23 (8.9)	0.264	1.46 (1.29–2.45)
	Relapse	9 (33.3)	6 (22.2)	10 (37.0)	2 (3.7)		

Linked by	PHF	45 (20.2)	65 (29.2)	103 (46.3)	19 (8.5)	0.868	5 (4.7–9.32)
	PPM	7 (17.9)	8 (20.5)	19 (48.7)	5 (12.8)		
	HEW	2 (15.3)	5 (38.4)	5 (38.4)	1 (7.6)		

TB type	PPOS	34 (24.2)	44 (31.4)	53 (37.8)	9 (6.4)	0.001	1.21 (1.09–2.7)
	PTB-	15 (25.4)	9 (15.2)	31 (52.5)	4 (6.7)		
	EP	5 (5.8)	25 (29.4)	43 (50.5)	12 (14.1)		

Weight group	<50	39 (28.6)	58 (42.6)	39 (28.6)	0 (0)	0.001	1.15 (0.91–2.13)
	50–60	14 (15.5)	18 (20.0)	56 (62.2)	2 (2.2)		
	>60	1 (1.7)	2 (3.44)	32 (55.1)	23 (39.6)		

Height group	<1.60	17 (16.6)	25 (24.50)	49 (48.0)	11 (10.7)	0.015	3.53 (1.79–4.51)
	1.60–1.75	22 (15.4)	44 (30.9)	67 (47.1)	13 (9.1)		
	>1.75	15 (41.6)	9 (25.0)	11 (30.5)	1 (2.7)		

HIV result	Positive	7 (13.7)	11 (21.5)	28 (54.9)	5 (9.8)	0.602	1.96 (1.47–2.7)
	Negative	47 (20.1)	67 (28.7)	99 (42.4)	20 (8.5)		

Household member	<3	35 (17.5)	57 (28.5)	97 (48.5)	11 (5.5)	0.019	15.75 (11.58–21.42)
	>3	17 (20.2)	21 (25.0)	32 (38.0)	14 (16.6)		

PHF, linked directly to the public health facility by themselves; PPM, linked to a TB clinic through public private members (NGOS and private clinics); HEW, linked to a TB clinic through a health extension worker; PPOS, bacteriologically confirmed pulmonary tuberculosis patient; PNEG, pulmonary tuberculosis patient with only chest radiography finding; EP, extrapulmonary tuberculosis patients. BMI, body mass index.

**Table 4:** Sociodemographic characteristic and change in body mass index (BMI) of tuberculosis patients 2 months after starting treatment.

Variable		No change in BMI, *N* (%)	Decrease in BMI, *N* (%)	Increase in BMI, *N* (%)	*p* value
All patients		35 (12.3)	65 (22.9)	183 (64.6)	

Age group	18–24	11 (17.2	7 (10.9)	46 (71.9)	0.002
	25–34	8 (37.6)	23 (21.9)	74 (70.4)	
	35–44	11 (25.5)	14 (32.5)	18 (41.8)	
	45–60	4 (10.0)	13 (32.5)	23 (57.5)	
	>60	1 (3.22)	8 (25.8)	22 (70.9)	

Sex	Male	21 (13.2)	37 (23.1)	102 (63.7)	0.394
	Female	14 (12.3)	33 (29.2)	76 (67.2)	

Treatment history	New	31 (12.1)	62 (24.2)	163 (63.6)	0.304
	Relapse	4 (14.8)	3 (11.1)	20 (74.0)	

Linked by	PHF	31 (13.4)	53 (22.9)	147 (63.6)	0.625
	PPM	4 (10.2)	8 (20.5)	27 (69.2)	
	HEW	0 (0)	4 (30.7)	9 (69.2)	

TB type	PPOS	13 (9.3)	31 (22.3)	95 (68.3)	0.126
	PTB-	7 (11.8)	19 (32.2)	33 (55.9)	
	EP	15 (17.6)	15 (17.6)	55 (64.7)	

Weight group	<50	16 (11.7)	27 (19.8)	93 (68.3)	0.321
	50–60	11 (12.3)	19 (21.3)	59 (66.2)	
	>60	8 (13.7)	19 (32.7)	31 (53.4)	

Height group	<1.60	13 (11.6)	23 (20.5)	66 (58.9)	0.902
	1.60–1.75	16 (11.0)	35 (24.1)	94 (64.8)	
	>1.75	6 (4.1)	7 (4.8)	23 (15.8)	

HIV result	Positive	5 (9.8)	15 (29.41)	31 (60.7)	0.713
	Negative	30 (12.9)	50 (21.5)	152 (65.8)	

Household member	<3	28 (14.0)	45 (22.6)	127 (63.3)	0.407
	>3	7 (8.3)	20 (23.8)	57 (67.85)	

BMI group	<16.0	5 (9.2)	6 (11.1)	43 (79.6)	0.001
	16.0–18.5	11 (14.1)	10 (12.8)	57 (73.0)	
	18.51–24.9	18 (14.2)	37 (29.3)	71 (56.3)	
	>24.9	1 (4.0)	12 (48.0)	12 (48.0)	

PHF, linked directly to the public health facility by themselves; PPM, linked to a TB clinic through public private members (NGOS and private clinics); HEW, linked to a TB clinic through a health extension worker; PPOS, bacteriologically confirmed pulmonary tuberculosis patient; PNEG, pulmonary tuberculosis patient with only chest radiography finding; EP, extrapulmonary tuberculosis patients; BMI, body mass index.

## Data Availability

The data sets used and/or analyzed during the current study are available from the corresponding author upon reasonable request.

## Abbreviations

AIDS: Acquired Immune Deficiency Syndrome
BMI: Body mass index
EPTB: Extrapulmonary tuberculosis
HEW: Health extension worker
HIV: Human Immunodeficiency Virus
MAM: Moderate acute malnutrition
PHF: Public Health Facility
PPOS: Pulmonary tuberculosis positive
PTB: Pulmonary tuberculosis negative
SAM: Severe acute malnutrition
TB: Tuberculosis.
